# Olfactory Ensheathing Cells Express α7 Integrin to Mediate Their Migration on Laminin

**DOI:** 10.1371/journal.pone.0153394

**Published:** 2016-04-14

**Authors:** Norianne T. Ingram, Rana R. Khankan, Patricia E. Phelps

**Affiliations:** Department of Integrative Biology and Physiology, University of California Los Angeles, Los Angeles, California, United States of America; School of Biomedical Sciences, The University of Queensland, AUSTRALIA

## Abstract

The unique glia located in the olfactory system, called olfactory ensheathing cells (OECs), are implicated as an attractive choice for transplantation therapy following spinal cord injury because of their pro-regenerative characteristics. Adult OECs are thought to improve functional recovery and regeneration after injury by secreting neurotrophic factors and making cell-to-cell contacts with regenerating processes, but the mechanisms are not well understood. We show first that α7 integrin, a laminin receptor, is highly expressed at the protein level by OECs throughout the olfactory system, i.e., in the olfactory mucosa, olfactory nerve, and olfactory nerve layer of the olfactory bulb. Then we asked if OECs use the α7 integrin receptor directly to promote neurite outgrowth on permissive and neutral substrates, *in vitro*. We co-cultured *α7*^*+/+*^ and α7^*lacZ/lacZ*^ postnatal cerebral cortical neurons with *α7*^*+/+*^ or α7^*lacZ/lacZ*^ OECs and found that genotype did not effect the ability of OECs to enhance neurite outgrowth by direct contact. Loss of α7 integrin did however significantly decrease the motility of adult OECs in transwell experiments. Twice as many *α7*^*+/+*^ OECs migrated through laminin-coated transwells compared to *α7*^*+/+*^ OECs on poly-L-lysine (PLL). This is in contrast to α7^*lacZ/lacZ*^ OECs, which showed no migratory preference for laminin substrate over PLL. These results demonstrate that OECs express α7 integrin, and that laminin and its α7 integrin receptor contribute to adult OEC migration *in vitro* and perhaps also *in vivo*.

## Introduction

Olfactory ensheathing cells (OECs) are unique and highly specialized glia. During embryonic development, OECs migrate with the axons of peripherally born olfactory receptor neurons (ORNs) into the olfactory nerve layer of the olfactory bulb. ORNs are continuously generated throughout life, and OECs play a key role in adult ORN turnover by assisting in the removal of degenerating neural remnants and guiding the replacement of new ORN axons [1, 2]. In addition to ensheathing ORN axons in the olfactory nerve and bulb, OECs also interact with the glia limitans, i.e., the astrocytic barrier that normally isolates the central from the peripheral nervous system [3]. Because of their abilities to support adult neuronal growth and cross the glia limitans, OECs are an attractive cell type for transplantation-based therapy following spinal cord injury [4–9].

At present the mechanisms by which OECs stimulate axonal outgrowth and regeneration are not well understood, but tissue culture experiments suggest that both growth factor secretion and cell-to-cell adhesion between OECs and neurites are involved [10–13]. Previous studies demonstrated that OEC secretion of brain-derived neurotrophic factor enhanced axonal outgrowth *in vitro* [12, 14]. OECs also secrete other neurotrophins that likely enhance neurite outgrowth, such as nerve growth factor, glial-derived neurotrophic factor, and ciliary neurotrophic factor [10, 15].

In addition to secreted factors, direct contacts between OECs and neurons have also increased neurite outgrowth and survival [11, 16]. To begin to understand how OEC-neurite contacts might stimulate neurite outgrowth by contact-mediated methods, we reviewed the cell adhesion molecules reportedly expressed by OECs. Some information regarding the nature of those contacts has come from microarray studies, which have indicated that olfactory bulb-derived OECs express many cell adhesion molecule candidates that could mediate OEC-neurite contact interactions, such as integrins α1, α6, and α7; cadherin 4; and neural cell adhesion molecules 2, 3 [17–20]. We focused on the laminin receptor α7β1 integrin for three reasons: 1) α7 expression is reported in regions of the olfactory bulb (OB) that contain OECs [21]; 2) Schwann cells express α7, and many characteristics are shared between these two types of glial cells, including their regenerative properties [22, 23]; and 3) α7 in Schwann cells reportedly regulates peripheral neurite outgrowth and regeneration [24–26].

In this study we asked if α7 integrin colocalizes with established OEC markers by taking advantage of a mouse line with a *lacZ* reporter inserted into exon 1 of the α7 integrin gene locus [27]. We also examined coexpression of dystroglycan (DG), because DG commonly colocalizes with α7 integrin in the nervous system and skeletal muscle [28]. To investigate the role of α7 integrin in OECs, we inquired if the deletion of α7 would affect neurite outgrowth in an OEC-neuron co-culture model, and if adult OECs use α7 to mediate their migration on laminin. Results from these experiments show that α7 integrin is a key-signaling molecule involved in the migration of OECs on a laminin matrix.

## Materials and Methods

### Animals and tissue preparation

All animal experiments were approved by the Institutional Animal Care and Use Committee of UCLA and were conducted in accordance with the National Institutes of Health *Guide for the Care and Use of Laboratory Animals*. The α7 integrin mouse line is on a C57BL/6J background and contains a *lacZ* reporter inserted into the first exon of the α7 integrin gene locus [27]. Heterozygous mice (*α7*^*lacZ/+*^*)* have one α7 allele, which supports normal function; whereas mutants (*α7* ^*lacZ/lacZ*^) express only β-galactosidase (β-gal). Results are described as α7/β-gal because β-gal localization is indicative of α7 integrin expression [27]. Pairs of *α7*^*lacZ/+*^ mice obtained from Drs. Dean Burkin and Rachelle Crosbie-Watson (Univ. of Nevada and UCLA) were bred to generate all three genotypes, which were then confirmed by PCR as described [27]. At weaning, males and females were separated and housed 5/cage with cotton nestlets. Breeding males were housed with 1–2 females or alone. Temperature of vivarium maintained at 72°±2°C.

Adult mice of either sex were deeply anesthesized with 100 mg/kg sodium pentobarbital and perfused transcardially with 4% paraformaldehyde followed by a 4 hr postfix. The olfactory bulbs and the attached nasal epithelium were dissected, cryoprotected, embedded, sectioned sagittally at 15–20 μm thickness, and slide mounted. Horizontal sections of the nasal epithelium also were slide mounted to examine cross sections of the olfactory nerve fascicles.

### β-Galactosidase histochemistry

Sections from all 3 genotypes were stained with X-gal solution (Gold Biotechnology, St. Louis, MO) as reported [29, 30]. X-gal was added to the sections for 4–5 hrs at 37°C. Strong blue staining was observed in sections from *α7*^*lacZ/+*^ and *α7*^*lacZ/lacZ*^ mice, but those from *α7*^*+/+*^ mice remained unstained.

### Immunohistochemistry

To confirm α7 integrin expression in adult OECs, *α7*^*lacZ/+*^ and *α7*^*lacZ/lacZ*^ olfactory bulbs and the associated olfactory epithelium were labeled with rabbit anti-α7 integrin or rabbit anti-β-gal and established OEC markers: SOX10, a neural crest nuclear marker [31]; S100β, a glia marker [1]; Aquaporin-1, a water channel [32], and brain lipid -binding protein (BLBP), an OEC marker [33]. Information on primary antibodies is listed in Table 1. Rabbit anti-β-gal immunohistochemistry was conducted first with either a standard immunofluorescent protocol, or if both primary antibodies were raised in rabbit, tyramide signal amplification (TSA; PerkinElmer, Waltham, MA). Standard fluorescent β-gal immunohistochemistry was carried out with 0.1M Tris + 1.4% NaCl + 0.1% BSA buffer (TBS). After 1 hr in 5% normal donkey serum (NDS), sections were left overnight in rabbit anti-β-gal (Table 1). Sections were washed in TBS, incubated for 1 hr in donkey anti-rabbit conjugated with Alexa 488 (1:200–250; Invitrogen, Grand Island, NY), and washed with TBS before beginning protocols with additional markers.

**Table 1:** Primary Antisera Used.

Primary Antisera	Immunogen	Source; Catalogue #	Host Species	Working Dilutions
**α7 integrin (ITGA7)**	Synthetic peptide within Human ITGA7 aa 730–780	Abcam (Cambridge, MA); AB203254	Rabbit polyclonal	1:100
**Aquaporin 1 (AQP1)**	19aa synthetic peptide (aa 251–269) from C-terminus	Chemicon (Temecula, CA); AB3065	Rabbit polyclonal	1:5,000
**β-dystroglycan (β-DG)**	15aa synthetic peptide from C-terminus of human β-DG	Developmental Studies Hybridoma Bank; MANDAG2 clone 7D11	Mouse IgG monoclonal	1:750
**β-galactosidase (β-gal)**	β-gal from E. coli.	MP Biomedicals (Solon, OH); 55976	Rabbit polyclonal	1:10,000 (IHC & TSA)
**β3-Tubulin**	Rat brain microtubules	Covance Laboratories (Emeryville, CA); PRB-435P	Rabbit polyclonal	1:1,500
**Brain lipid-binding protein (BLBP)**	GST-tagged recombinant protein corresponding to human BLBP	EMD Millipore; AB9558	Rabbit polyclonal	1:1,500
**Laminin α1**	Mouse sarcoma	Sigma; L9393	Rabbit polyclonal	1:2,500–5,000
**Olfactory marker protein (OMP)**	Rodent OMP	Wako (Richmond, VA); 544–10001	Goat polyclonal	1:5,000
**p75 nerve growth factor receptor (p75)**	Extracellular fragment from third exon in mouse p75 (aa 43–161)	Chemicon (Millipore, Billerica, MA); AB1554	Rabbit polyclonal	1:1,500–2,000 (OEC panning) 1:5,000 (IHC)
**SOX10**	E. coli-derived recombinant human SOX10	R&D Systems (Minneapolis, MN); AF2864	Goat polyclonal	1:75
**S100β**	S100β from cow brain	Dako A/S (Glostrup, Denmark); Z0311	Rabbit polyclonal	1:30,000

Standard immunofluorescent protocols were used with the following antibodies: goat anti-SOX10, goat anti-OMP (a marker for mature olfactory receptor neurons), rabbit anti-laminin α1, rabbit anti-β3 tubulin, rabbit anti-α7 integrin, rabbit-anti-BLBP and/or mouse anti-β-DG (which recognizes the β subunit of the dystroglycan complex). Sections were rinsed with 0.1M phosphate buffered saline (PBS). They were then blocked with 5% NDS and incubated in primary antibodies overnight. After rinsing, sections were placed in donkey anti-goat secondary conjugated with Alexa Fluor 594 (1:800; Invitrogen) or Alexa Fluor 488 (1:250–500; Invitrogen) for 1 hr, rinsed, and coverslipped with Fluorogel (EMS, Hatfield, PA).

If both primary antibodies were generated in rabbit, the TSA fluorescein kit and TSA methods were used as reported [29, 32]. Between the two TSA protocols, sections were fixed for 15 min with 4% paraformaldehyde and treated with citric acid at pH 6.0 [32, 34]. Controls for these double TSA experiments included elimination of the second primary to assess incomplete inactivation of the first primary with the citric acid treatment. To amplify the anti-S100 and anti-AQP1 signals the TSA protocol was used with TBS buffer. After primary incubation, sections were incubated in donkey anti-rabbit IgG (1:500) for 1 hr, followed by streptavidin conjugated to Alexa 594 (1:1200, Invitrogen).

### Dissociated OEC cultures

Primary OEC cultures were generated from olfactory bulbs dissected from two 8–10 week-old mice per genotype with methods adapted from [12, 35]. DMEM/F12 (D/F) medium with 15% fetal bovine serum (FBS) and 1% Penicillin/Streptomycin (Gibco, Rockville, MD) was changed daily. After 5–6 days *in vitro*, primary OEC cultures were immunopurified with rabbit anti-p75-NGFR (1:1500–2000) and seeded in 4-chamber polystyrene culture slides (BD Falcon, San Jose, CA), coated with poly-L-lysine (PLL; 25 mg/ml; Sigma, St. Louis, MO) to characterize the cell types in the cultures and provide the cellular substrate for neuron-OEC co-cultures. Cell Tracker Green (7 μM in serum-free D/F media; Invitrogen) was added to wells with OECs for 1 hr and then activated with D/F + 15% FBS incubation for 1 hr.

### Co-culture assay and analysis

To assess the contribution of α7 integrin to neurite outgrowth, we generated OEC-cortical neuron co-cultures in triplicate with 2 wells per variable during each of the three culture dates. Neurons from postnatal day 7–8 mice were dissected from both *α7*^*+/+*^ and *α7*^*lacZ/lacZ*^ cerebral cortices and centrifuged in an iodixanol step-gradient gel to enrich for neurons (Optiprep; Sigma) [36]. Cortical neurons (50-100K) from each genotype were seeded on 4-chamber culture slides with the following variables: 1) PLL covered with laminin (10 μg/ml; Invitrogen), 2) PLL alone (25 mg/ml), 3) PLL with *α7*^*+/+*^ OECs, and 4) PLL with *α7*^*lacZ/lacZ*^ OECs.

Co-cultures were incubated for 36 hrs at 37°C with 5% CO_2_ and then fixed for 10–15 min with 4% paraformaldehyde. Neurons were identified with rabbit anti-β3-tubulin (Table 1) and standard methods of immunolocalization. We photographed 10 fields that contained the most cells from each culture well with the 20x objective of an Olympus microscope. All neurons were traced within each field, and neurites were measured with Neurolucida and Neurolucida Explorer software (MBF Bioscience, Williston, VT). Individual neurites were scored on the type of interaction with OECs adapted from Khankan et al. [16]. Neurite and OEC interactions were defined as: 1) aligning with each other, 2) crossing each other, or 3) having no contact. Analyses focused on total neurite length by genotype and individual neurite length relative to their OEC interaction. Significance of means was evaluated by a two-way analysis of variance (ANOVA). Calculations were carried out in JMP 9.0 (SAS Institute, Cary, NC).

### Migration assay and analysis

To test if adult OECs require α7 integrin for migration on a laminin substrate, transwell inserts (0.8 μm pore, 24-well; BD Falcon) were coated with either PLL alone (25 mg/ml) or PLL coated with laminin (10 μg/ml). Migration experiments were repeated 4 times with 2 inserts per group. Experimental groups were: 1) *α7*^*+/+*^ OECs, 2) *α7*^*lacZ/+*^ OECs, and 3) *α7*^*lacZ/lacZ*^ OECs, with each genotype grown on PLL with laminin or PLL alone. Inserts were placed in 24-well plates with 400 μl of D/F medium plus 15% FBS beneath the insert to stimulate migration [37]. Purified OECs were added to the top of the transwell insert in serum-free D/F medium (20–25,000 cells/300 μl) and placed in a 37°C incubator with 5% CO_2_. After 24 hrs 200 μl of media was removed from each well, and replaced with 400 μl of fresh D/F media with 15% FBS to further stimulate migration by increasing the serum gradient. After 48 hrs, the top of each insert was scraped to remove non-motile cells, and the insert was fixed with 4% paraformaldehyde for 10 min. OECs on the bottom of the inserts were identified with rabbit anti-p75 NGFR (Table 1) and the Hoechst nuclear marker (1:300, Sigma). Entire inserts were photographed at 4x, and all OEC nuclei were counted (800–9000 cells/insert) with Neurolucida software (MBF Bioscience).

We normalized our cell counts in the following way. The number of OEC nuclei migrating on PLL with laminin were counted and divided by the number of nuclei with the same genotype migrating on PLL alone; we called this ratio the “migratory-potential ratio.” Thus, a migratory-potential ratio of 1 represents equal migration on PLL alone and PLL coated with laminin. Significance of the mean migratory potential was evaluated with a one-way ANOVA using the JMP 9.0 program.

### Photography

Fluorescent and bright field images were taken on an Olympus microscope or a Zeiss LSM 510 confocal microscope. Confocal images represent 1–3 μm-thick Z-stacks collected with either 25x or 63x oil objectives or a 40x water-corrected objective. Images were assembled with Adobe Photoshop and modified only to match exposure levels for comparisons.

## Results

### α7 integrin localizes to OEC-rich areas in the primary olfactory system

**Fig 1:**
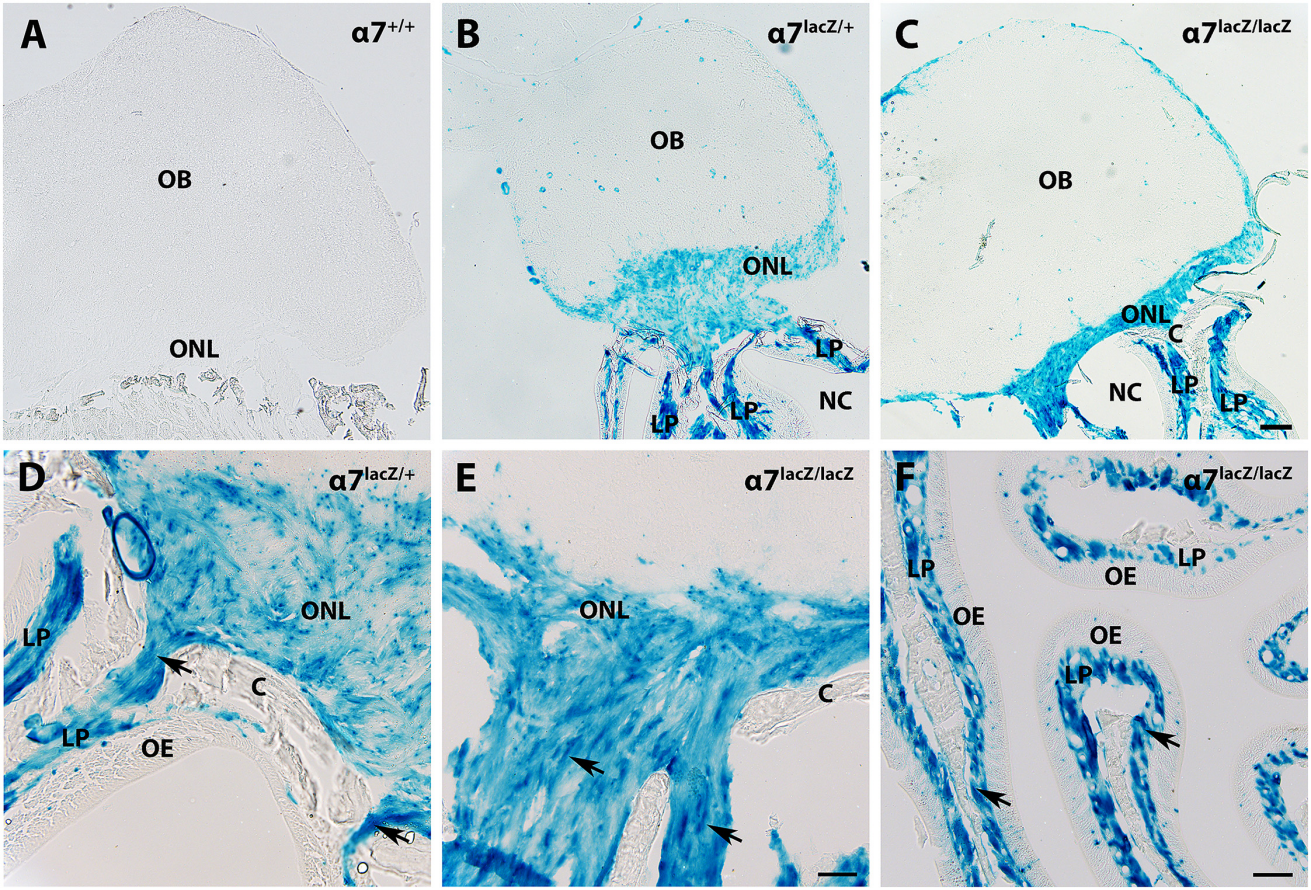
OEC-rich areas of the olfactory system express β-galactosidase (β-gal) in *α7*^*lacZ/+*^ and *α7*^*lacZ/lacZ*^ mice. A: In the central (olfactory bulb, OB) and peripheral (nasal cavity, NC) olfactory areas, sagittal sections from *α7*^***+/+***^ mice have no β-gal reaction. B, C: Mice with one (*α7*^*lacZ/+*^, B) or two copies (*α7*^*lacZ/lacZ*^, C) of the *α7*^*lacZ*^ allele have similar patterns of β-gal histochemistry. *α*7/β-gal is within the olfactory nerve layer (ONL) and the lamina propria (LP), two areas heavily populated with OECs. Blood vessels also express *α*7/β-gal. D, E: Arrows point to the β-gal-labeled olfactory nerve (cranial nerve I) as it courses through the cribriform plate (C) that separates the nasal cavity from the olfactory bulbs. F: A horizontal section of *α7*^*lacZ/lacZ*^ olfactory mucosa shows intense β-gal expression in the LP (arrows) and light reactivity in the olfactory epithelium (OE). Scale bars for A-C: 200 μm; D-E: 50 μm; F: 100 μm.

We first examined α7 integrin expression in the olfactory system with X-gal histochemistry. The olfactory bulb sizes appeared similar among the three α7 genotypes. Coronal sections of both nasal mucosa and olfactory bulbs showed no β-gal reaction product in *α7*^*+/+*^ controls (Fig 1A) compared to an intense blue, β-gal reaction product in the nasal lamina propria and olfactory nerve layer (ONL) of the *α7*^*lacZ/+*^ and *α7*^*lacZ/lacZ*^ olfactory bulbs (Fig 1B and 1C). The olfactory nerve also expressed α7 integrin (Fig 1D and 1E, first cranial nerve, indicated by arrows), as the nerve coursed from the lamina propria into the ONL that surrounds the bulb (Fig 1B and 1C). The majority of the olfactory epithelium is β-gal-negative compared with the strongly labeled lamina propria (Fig 1F). β-gal precipitate also associated with blood vessels presumably from α7 integrin on vascular smooth muscle [27, 38].

**Fig 2:**
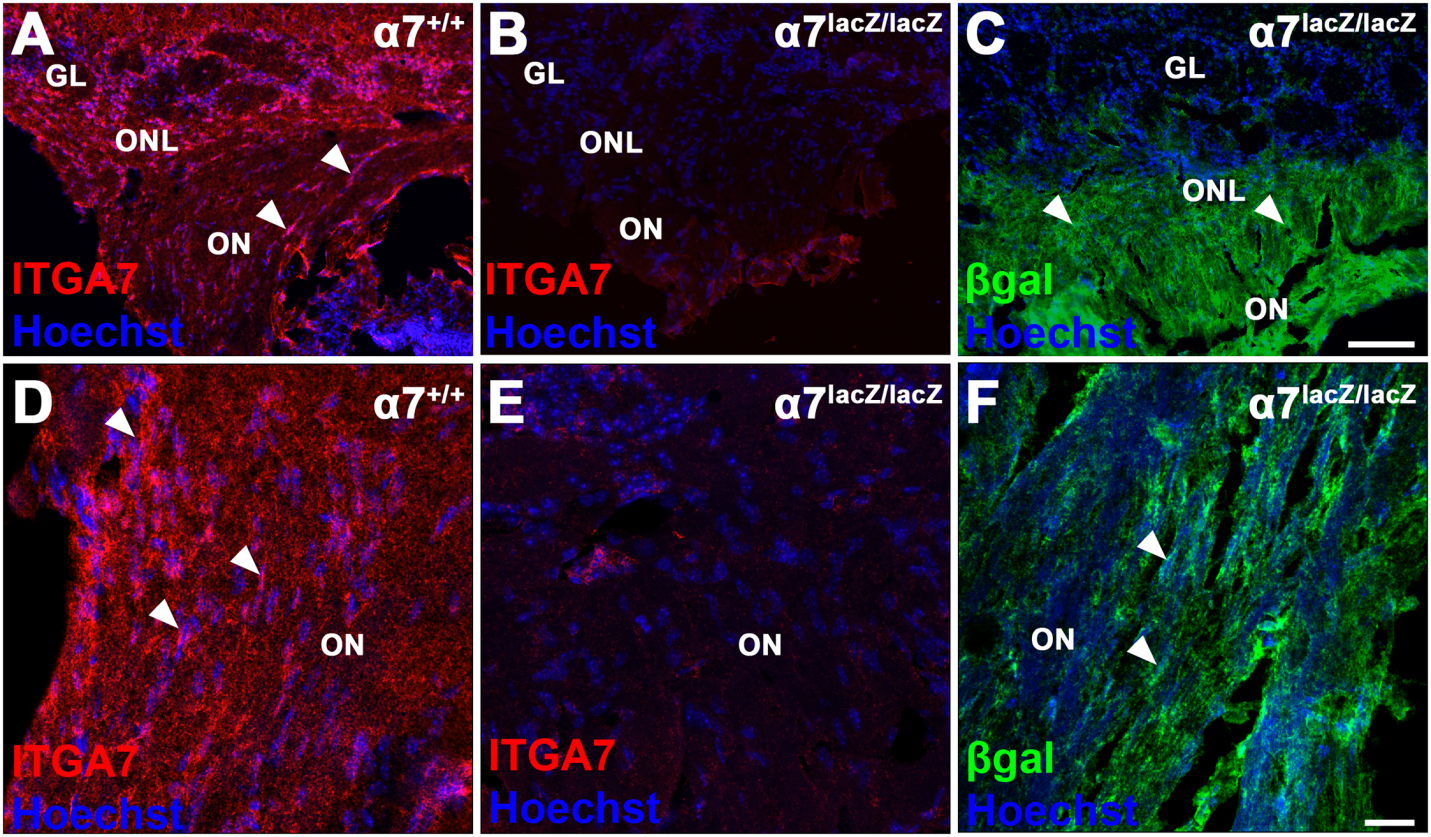
Expression of α7 integrin and β-gal in the olfactory nerve. A-C: Anti-α7 integrin (ITGA7, red) and Hoechst nuclear stain (blue) show the presence of α7 protein in *α7*^*+/+*^ (A) but not *α7*^*lacZ/lacZ*^ (B) olfactory nerve (ON, arrowheads), olfactory nerve layer (ONL) and glomerular layer (GL). Adjacent *α7*^*lacZ/lacZ*^ section (C) has *α*7/β-gal reactivity (green) in the ON and ONL (arrowheads). D-F: OECs in the ON are marked with arrowheads and show antibody expression in *α7*^*+/+*^ (D) or *α*7/β-gal in *α7*^*lacZ/lacZ*^ nerve (F). The *α7*^*lacZ/lacZ*^ negative control shows only Hoechst-labeled nuclei (E). Scale bar A-C: 100 μm; D-F: 20 μm.

Next we used antibodies to confirm the presence of α7 integrin in the olfactory system. α7 expression is present in *α7*^*+/+*^ and absent in *α7*^*lacZ/lacZ*^ olfactory nerve and bulb (Fig 2A, 2B, 2D and 2E). *α*7/β-gal reactivity is distributed within the *α7*^*lacZ/lacZ*^ olfactory nerve and ONL (Fig 2C and 2F). The *α7*^*+/+*^ glomerular layer contains *α*7 integrin, but the circular glomeruli are unstained (Fig 2A). Levels of β-gal expression in the *α7*^*lacZ/lacZ*^ glomerular layer are low (Fig 2C). From these results, we conclude that the areas of the olfactory system reported to contain OECs [1, 39] encompass the vast majority of the β-gal and antibody reaction products, and the deletion of α7 integrin does not cause gross anatomical defects in the lamina propria or ONL.

### α7/β-gal expression is found on OECs

**Fig 3:**
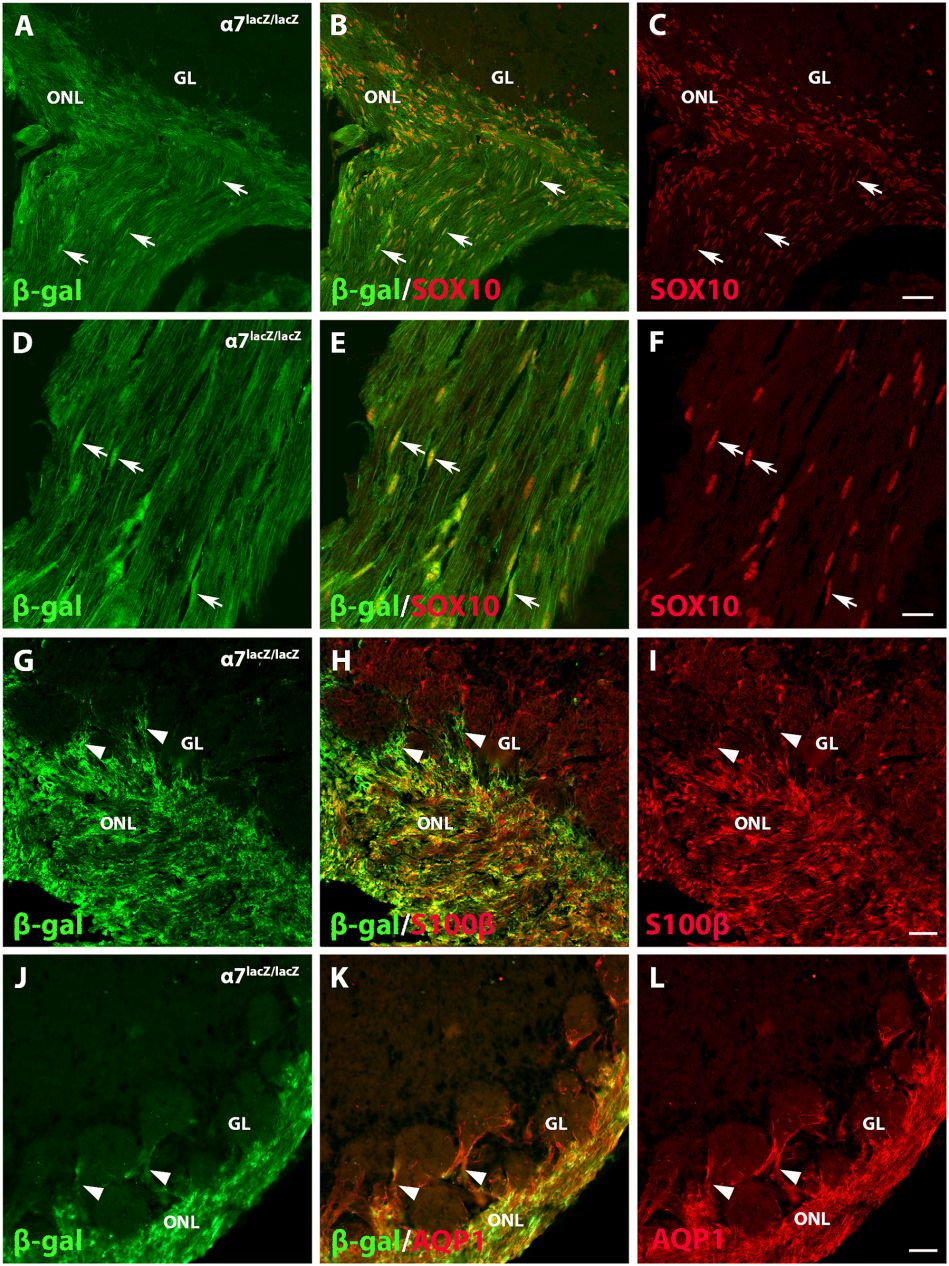
α7 integrin colocalizes with OEC markers SOX10, S100β, and AQP1 in the olfactory bulb. A-C: Expression of α7/β-gal (green) in *α7*^*lacZ/lacZ*^ olfactory bulbs overlaps with SOX10 expression (red nuclei) at arrows. D-F: A confocal image (4.0 μm-thick Z-stack) of the olfactory nerve shows α7/β-gal immunofluorescence (green) within the OEC cell bodies (arrows) and their thin processes. SOX10 (red) and β-gal staining colocalize within OEC nuclei (arrows). G-I: The α7/β-gal product (green) appears to colocalize in the *α7*^*lacZ/lacZ*^ olfactory nerve layer (ONL) with the glial marker S100β (red). OEC processes extend into the glomerular layer (GL; arrowheads) but don’t enter the glomeruli. J-L: The water channel Aquaporin 1 (AQP1, red) is highly expressed in the ONL and colocalizes with α7/β-gal fluorescence (green). Arrowheads mark AQP1 and light β-gal expression around but not within the glomeruli, the typical distribution of OECs. Scale bars for A-C, G-L: 50 μm; D-F: 20 μm.

To establish that α7 integrin is expressed in OECs, we colocalized α7/β-gal with three established OEC markers: SOX10, S100β, and AQP1. In *α7*^*+/+*^ olfactory bulbs, OEC markers stained only the olfactory nerve and ONL (data not shown). The *α7*^*lacZ/lacZ*^ olfactory bulbs and olfactory nerve fascicles, however, showed overlapping expression of α7/β-gal in OEC cell bodies and SOX10-positive nuclei (Fig 3A–3F). The stronger expression of α7/β-gal within the olfactory nerve outlines the nuclei and adjacent cytoplasm of OECs (Fig 3D, arrows), whereas the lighter immunoreactivity marks their thin processes (Fig 3D and 3E). SOX10 labels the OEC nuclei and colocalizes with the intense α7/β-gal reactivity (Fig 3E and 3F).

Previous studies showed that S100β is expressed by OECs within the olfactory nerve, the ONL, and the glomerular layer, as well as by astrocytes throughout the olfactory bulb [1, 32]. We found that in *α7*^*lacZ/lacZ*^ olfactory bulbs, α7 integrin expression was restricted to the ONL and processes that enter the glomerular layer, but it was not found within glomeruli, a pattern that is characteristic of OECs (Fig 3G–3I; [1, 32]). S100β is more broadly expressed but does colocalize with much of the α7/β-gal in the ONL and in OEC processes entering the glomerular layer (Fig 3G–3I, at arrowheads). The water channel AQP1 labels only OECs within the olfactory system [32], and α7/β-gal is coexpressed with AQP1 within the ONL and OEC processes around the glomeruli (Fig 3J–3L, at arrowheads). In combination, these results imply that peripheral and central OECs both express α7 integrin.

### Mature olfactory receptor neurons do not express α7 integrin

**Fig 4:**
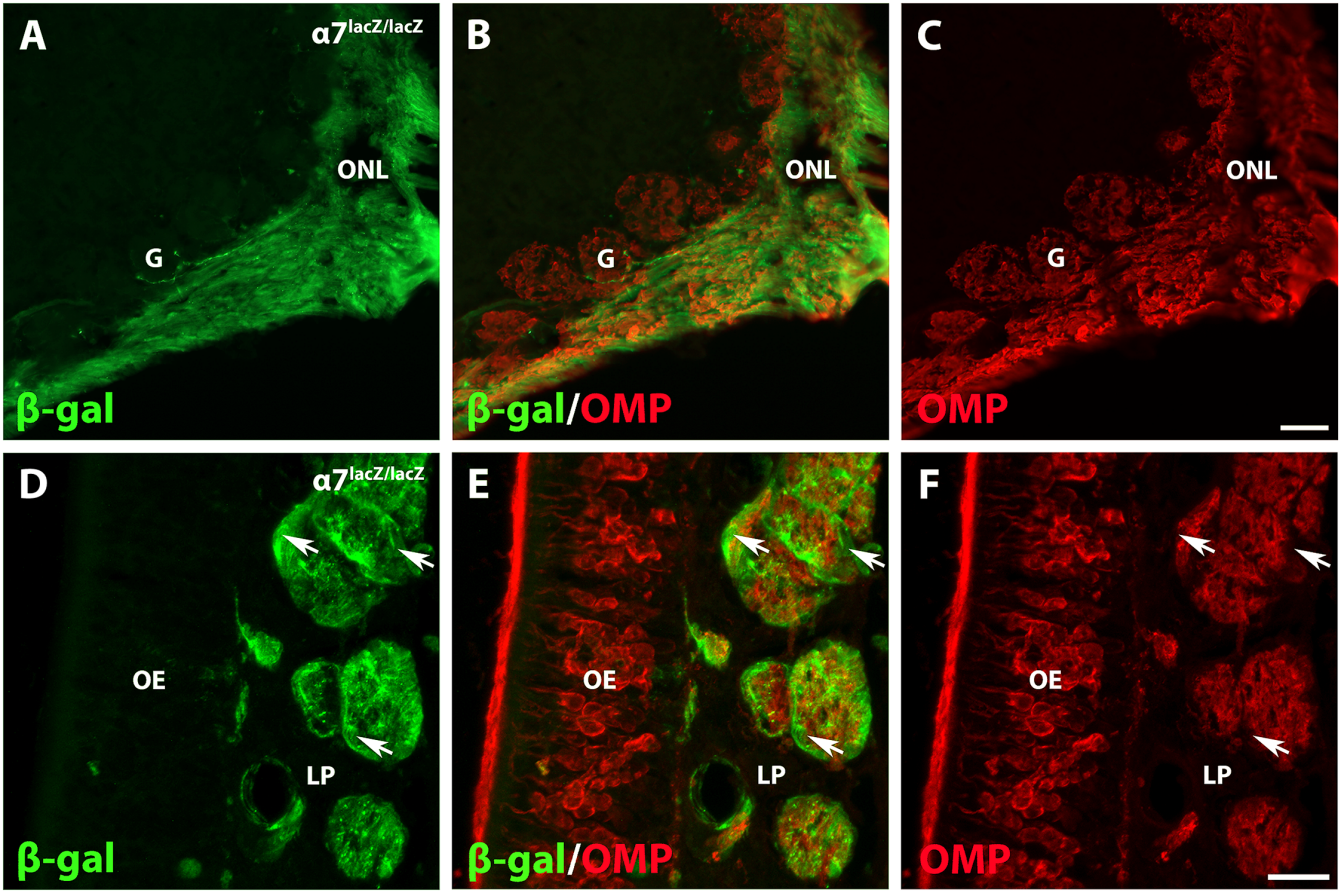
α7/β-gal is not expressed by mature olfactory receptor neurons. A-C: The *α7*^*lacZ/lacZ*^ olfactory bulb expresses α7/β-gal (green) in the olfactory nerve layer (ONL), and is closely associated with the olfactory marker protein (OMP, red). In axons of mature olfactory receptor neurons, OMP immunoreactivity fills their synaptic target, the glomeruli (G). α7/β-gal expression and OECs are excluded from the glomeruli. D-F: Fascicles of the olfactory nerve within the lamina propria (LP) have distinct areas of β-gal (green, arrows) and OMP-positive (red) immunoreactivity. β-gal-labeled OECs surround the OMP-labeled axon bundles. The olfactory epithelium (OE) contains OMP-labeled (red) olfactory receptor neurons. Scale bars for A-C: 50 μm; D-F: 20 μm.

To exclude the possibility that α7 integrin is expressed by axons of mature olfactory receptor neurons (ORNs), we colocalized α7/β-gal with the olfactory marker protein (OMP). As depicted in Fig 4C and 4F, ORNs expressed OMP in cell bodies residing in the olfactory epithelium as well as in axons of the lamina propria, ONL, and in their synaptic targets, the glomeruli (G; [40]). In *α7*^*lacZ/lacZ*^ olfactory bulbs, α7/β-gal was closely associated with OMP-labeled processes but each marker displayed a distinct pattern. Specifically, axons that express OMP entered the circular glomerular neuropil of the olfactory bulb, but α7/β-gal-labeled OECs did not (Fig 4A–4C). In the lamina propria, bundles of OMP-labeled axons are surrounded by α7/β-gal (Fig 4D–4F, arrows; [41]). These results confirm that α7 integrin is restricted to the glial compartment of the olfactory system that ensheath the axons of the ORNs, which are the OECs.

### β-dystroglycan is found in the primary olfactory system

**Fig 5:**
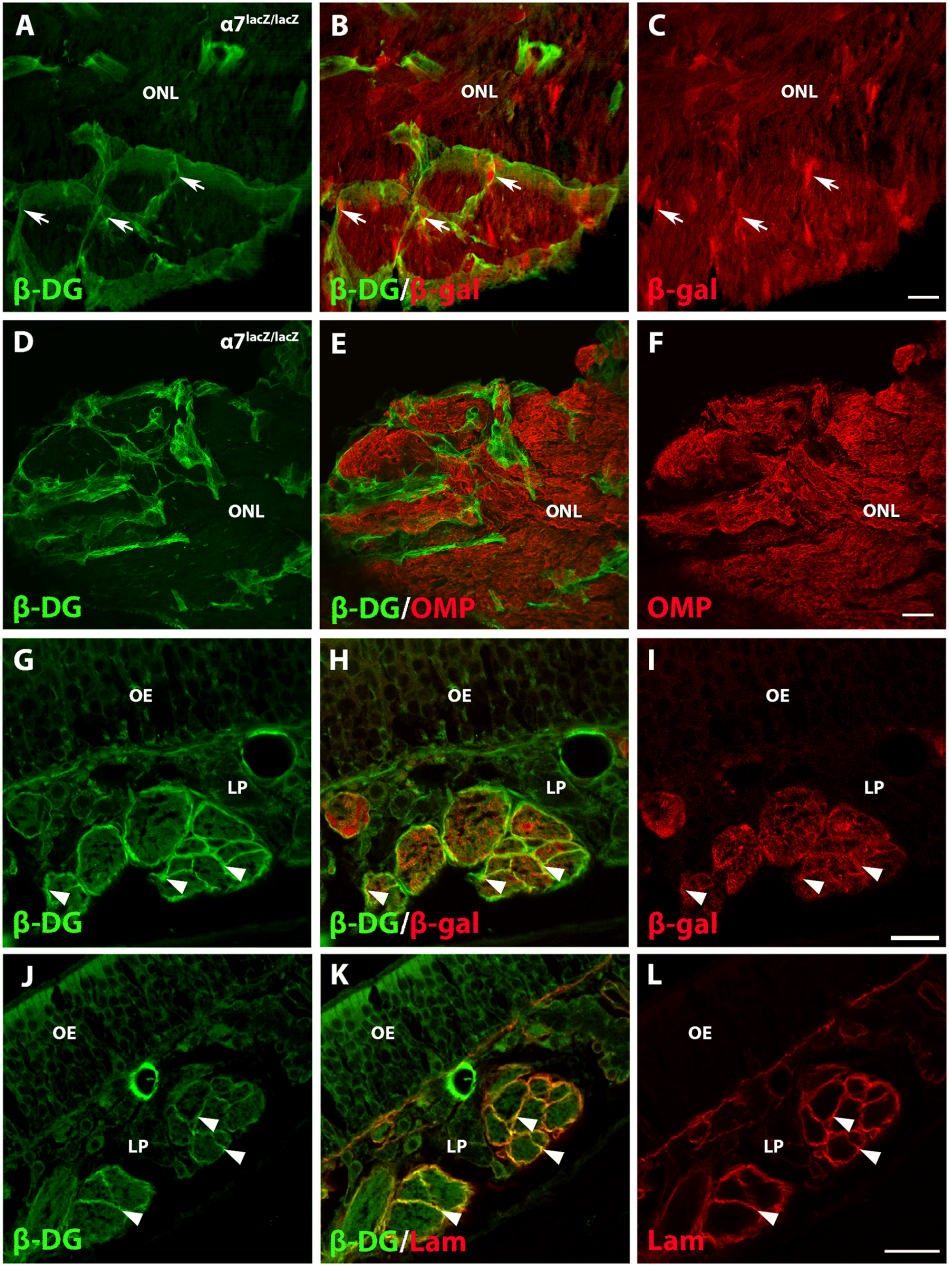
β-Dystoglycan is expressed on the outer OEC membrane. A-C: In the ONL of the olfactory bulb, β-dystroglycan (β-DG, green) is concentrated on the perimeters of large nerve fascicles, whereas α7/β-gal (red) is distributed broadly. α7/β-gal strongly stains OEC cell bodies (arrows) while β-DG immunoreactivity is restricted to the outer OEC membranes. D-F: In the ONL, β-DG reactivity is excluded from mature olfactory receptor neurons identified with OMP (red). G-I: In the nasal cavity β-DG (green) and α7/β-gal (red) are both strongly expressed around the perimeter of cross sections of olfactory nerve fascicles (arrowheads). J-L: Laminin α1 reactivity (red) colocalizes with β-DG on olfactory nerve fascicles. Scales bars for A-C, D-F, G-I, and J-L: 20 μm.

In skeletal muscle and Schwann cells, both α7 integrin and the DG complex are expressed together and form receptors for laminin in basement membranes [23, 42, 43]. Additionally, Roet et al. [19] reported that OECs express the mRNA for DG 1 and Takatoh et al. [44] demonstrated β-DG expression on the outer membrane of wildtype OECs but not on their inner leaflets. We therefore asked if *α7*^*lacZ/lacZ*^ OECs also express the β-DG subunit. Large fascicular structures were immunopositive for β-DG in the olfactory nerve and outer ONL of the olfactory bulb (Fig 5A and 5B, arrows; S1A and S1B Fig, arrowheads). The α7/β-gal OEC cell bodies that interdigitated within the fascicle appeared to lack β-DG expression (Fig 5A–5C, arrows; S1A–S1C Fig, arrowheads). The green β-DG reactivity also was distinct from the pattern of OMP expression in the olfactory bulb, indicating that ORNs do not express β-DG (Fig 5D–5F) [44].

The nasal mucosa also contained extensive β-DG expression around the olfactory nerve fascicles (Fig 5G, 5H, 5J and 5K). Mice with the α7^*lacZ*^ reporter showed β-DG and α7/β-gal spanning the outer membrane, whereas only α7/β-gal reactivity appeared on the inner leaflets of OEC fascicles (Fig 5G–5I, arrowheads). To exclude the possibility that β-DG is expressed on perineural fibroblasts adjacent to OEC-ORN fascicles, we colocalized laminin α1 with β-DG (Fig 5J–5L). Laminin immunoreactivity colocalized extensively with the β-DG staining (Fig 5K, arrowheads), a finding consistent with the interpretation that β-DG is expressed on OEC outer membranes.

### Does α7 integrin enhance OEC-induced neurite outgrowth?

**Fig 6:**
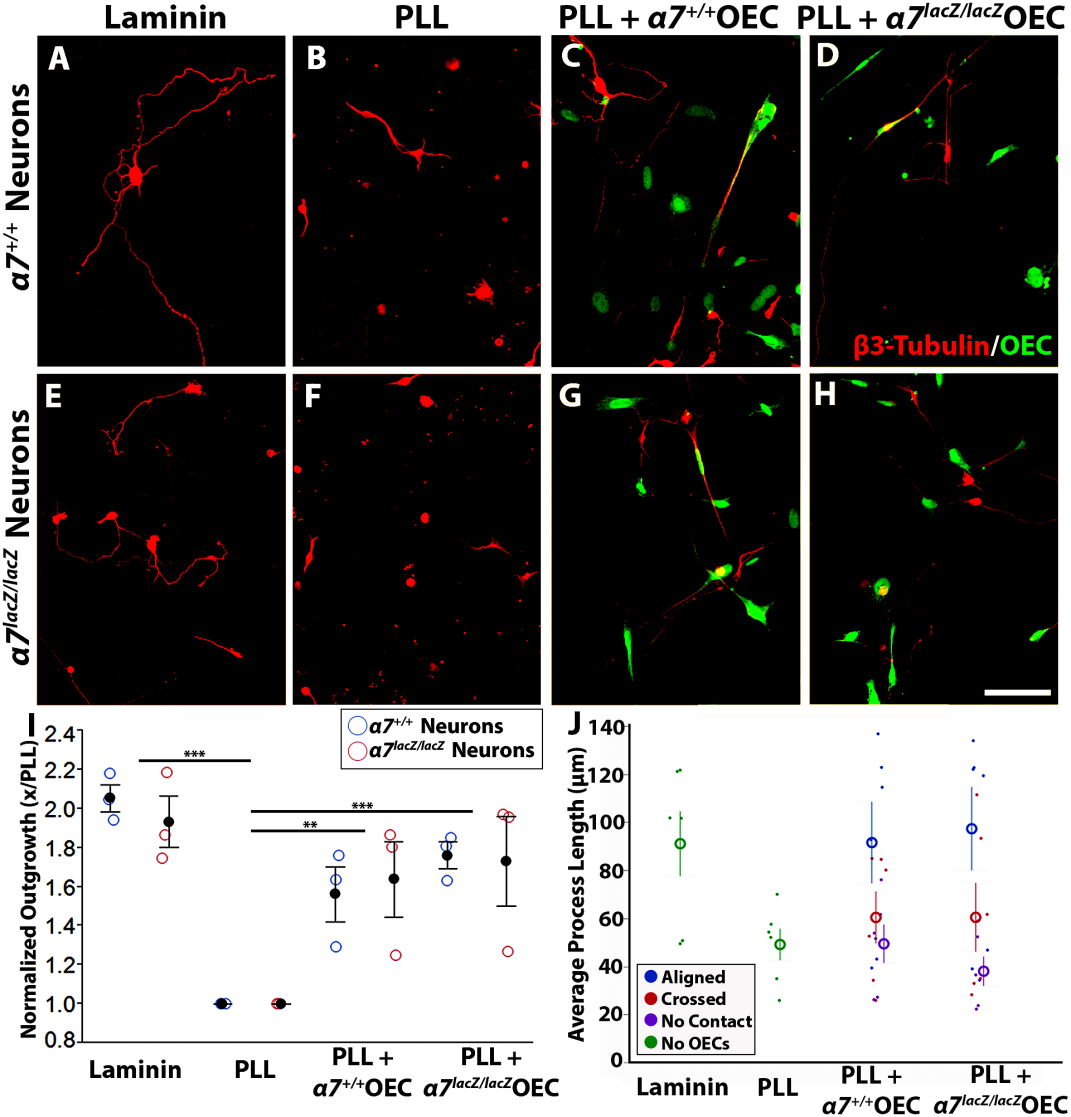
α7 integrin does not facilitate OEC-induced neurite outgrowth. A-H: Representative images from the experimental groups. A-D show cultures seeded with *α7*^*+/+*^ cortical neurons, whereas E-H contained *α7*^*lacZ/lacZ*^ neurons. Neurons were cultured on laminin (A, E), PLL (B, F), PLL + *α7*^*+/+*^ OECs (C, G), and PLL + *α7*^*lacZ/lacZ*^ OECs (D, H). Neurons were visualized with β3-tubulin, and OECs marked with Cell Tracker Green. I: Total neurite outgrowth per well was normalized to culture-matched outgrowth on PLL (normalized mean ± SEM is plotted; See S1 Table for raw data). Neuronal genotype did not affect neurite outgrowth on laminin or PLL (n = 3, *p* = 0.838). The laminin substrate induced more neurite growth than PLL (1.99 ± 0.07 units normalized; ***p<0.0001), and OECs enhanced neurite outgrowth lengths at a level that did not differ from laminin. The addition of OECs significantly increased the neuronal outgrowth compared to PLL only levels (*α7*^*+/+*^ OECS: 1.60 ± 0.11 **p = 0.0003; *α7*^*lacZ/lacZ*^ OECs: 1.71 ± 0.14, ***p<0.0001). J: For neuron-OEC co-cultures we sorted individual neurite lengths by the type of association they made with OECs (i.e., aligned, cross, no contact) and averaged them. The lengths of neurites that aligned with OECs were compared to neurites grown on laminin, PLL, and different OEC associations. (See S2 Table for averages, further description, and *p*-values). Scale bar A-H: 50 μm.

OECs promote neurite outgrowth by both secreted factors [11, 12, 14, 15] and cell-mediated contact [11, 16]. To test if α7 integrin is required for or enhances neurite outgrowth, we cultured OECs from olfactory bulbs of *α7*^*+/+*^ and *α7*^*lacZ/lacZ*^ mice. The cultured *α7*^*+/+*^ and *α7*^*lacZ/lacZ*^ OECs appeared similar in morphology. After immunopurification, the secondary cultures contained 86 ± 9% p75-NGFR-labeled OECs. Neurons from *α7*^*+/+*^ and *α7*^*lacZ/lacZ*^ mouse cerebral cortices at postnatal day 7–8 were prepared and seeded on the following substrates: 1) laminin, the extracellular matrix ligand that binds α7 integrin and our positive control (Fig 6A and 6E); 2) PLL, a neutral substrate and control (Fig 6B and 6F); and 3) PLL together with either *α7*^*+/+*^ OECs or *α7*^*lacZ/lacZ*^ OECs (Fig 6C, 6D, 6G and 6H).

To evaluate the average total neurite growth per neuron on each substrate, we measured and summed total neurite outgrowth and then divided this sum by the number of neurite-bearing neurons (S1 Table). Because the neutral PLL substrate produced a baseline level of outgrowth (Fig 6B and 6F), total neurite lengths per neuron were normalized for each substrate by dividing by the genotype-matched value from PLL cultures. No differences in total neurite length were detected between *α7*^*+/+*^ and *α7*^*lacZ/lacZ*^ cortical neurons grown on the same substrate (Fig 6I, S1 Table). Therefore, data generated with *α7*^*+/+*^ and *α7*^*lacZ/lacZ*^ neurons were combined for subsequent analysis.

Next we determined if the normalized neurite outgrowth on the four substrates differed. Total neurite outgrowth on laminin was twice that found on PLL (laminin, 2.0 ± 0.07 units normalized, versus PLL, 1.0 ± 0, mean ± SEM, n = 3; *p*<0.0001). When neuronal outgrowth on laminin was compared with that from OECs with or without α7 integrin, there were no significant differences (S1 Table). By contrast, outgrowth on PLL alone was substantially less than when *α7*^*+/+*^ or *α7*^*lacZ/lacZ*^ OECs were added to PLL (*α7*^*+/+*^ OECs: 1.6 ±0.11, n = 3; *p* = 0.0003; *α7*^*lacZ/lacZ*^ OECs: 1.7 ± 0.14, n = 3; *p*<0.0001). There were no differences, however, in outgrowth of neurites seeded on *α7*^*+/+*^ or *α7*^*lacZ/lacZ*^ OECs.

Finally, to evaluate if direct neurite interactions with OECs are responsible for enhanced outgrowth, the length of individual neurites was analyzed relative to their interactions with OECs. Individual neurites were categorized as directly aligned with, crossed, or not associated with OECs [16]. Our data showed that neurites that aligned with OECs were significantly longer than those that did not contact OECs (S2 Table). In fact, neurites that align with OECs had processes that were similar in length to those grown on the permissive laminin substrate (S2 Table). Likewise, the lengths of neurites that did not associate with glia did not differ from those grown on PLL alone (S2 Table). No differences were observed in outgrowth between aligned neurites from the *α7*^*+/+*^ or *α7*^*lacZ/lacZ*^ OEC cultures. Together these results suggest that loss of α7 integrin in *α7*^*lacZ/lacZ*^ mice does not hinder the enhancement of process outgrowth produced by cell-to-cell contact between OECs and neurites.

### Does α7 integrin function in adult OEC motility?

**Fig 7:**
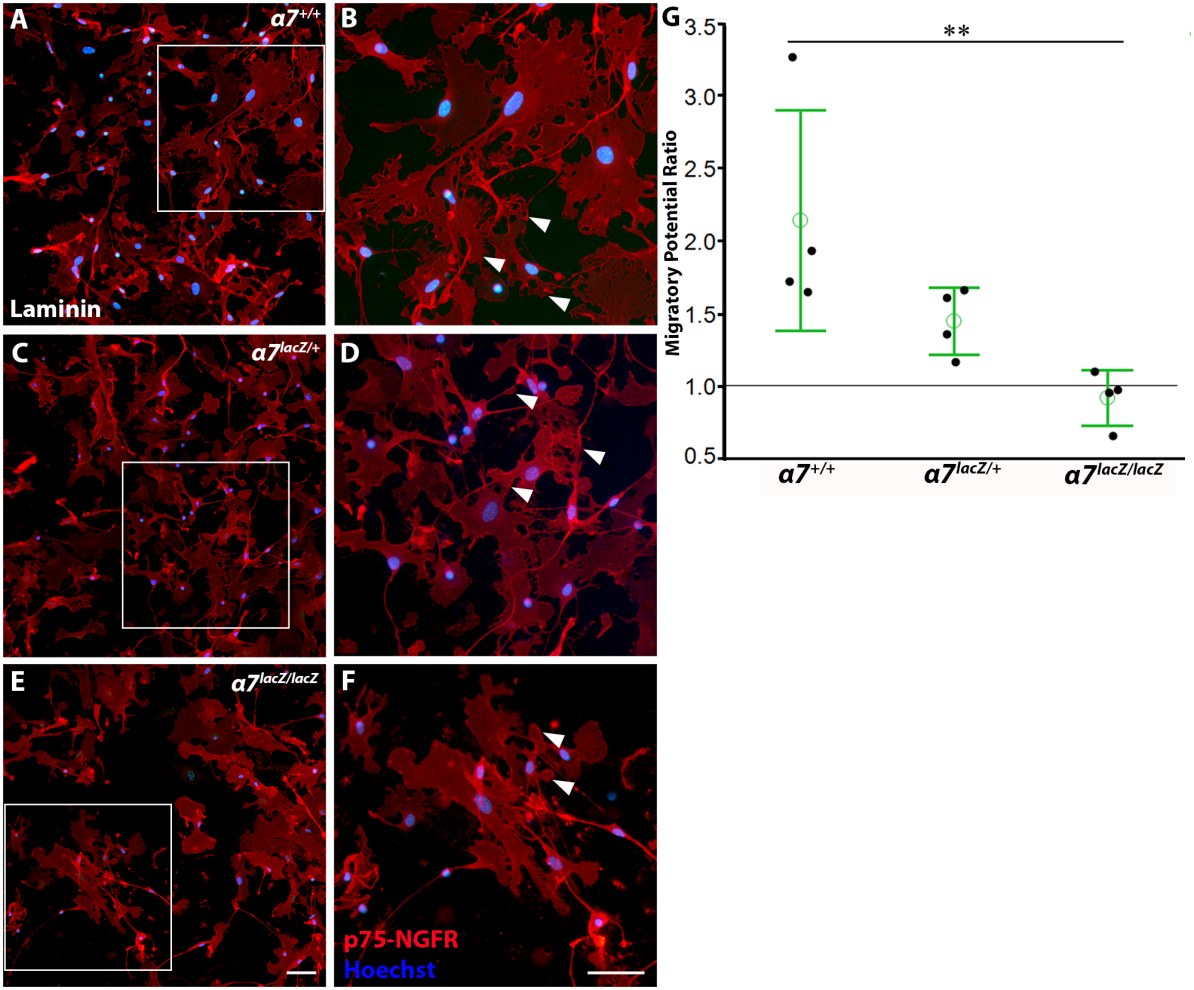
α7 integrin mediates OEC migration on a laminin substrate. A-F: Immunopurified OECs from all three genotypes migrated through transwell inserts coated with laminin. Enlargements of boxed areas A, C, and E are shown in B, D, and F. OECs have numerous membrane protrusions (arrowheads) that interact with other OECs. G: The migratory-potential ratio was used to assess the number of p-75-NGFR-labeled OECs that migrated through the laminin-coated insert divided by those that migrated on PLL-coated inserts. The mean migratory-potential ratio for *α7*^*+/+*^ OECs was 2.14 ± 0.76, and for *α7*^*lacZ/+*^ OECs was 1.45 ± 0.23; *α7*^*lacZ/lacZ*^ OECs did not have a substrate preference (0.92 ± 0.19). Many more *α7*^*+/+*^ OECs migrated through the laminin-coated transwell membranes than *α7*^*lacZ/lacZ*^ OECs (**p<0.01). Scale A, C, E: 50 μm; B, D, F: 50 μm.

OEC migration was evaluated on transwell membrane inserts coated with PLL or PLL with laminin; OECs were stimulated to migrate by adding 15% FBS beneath the inserts [30, 37]. After fixation, the OECs that migrated through laminin-coated membranes were visualized with anti-p75-NGFR and shown to have extensive membrane protrusions that contacted other OECs (Fig 7A–7F, arrowheads). The OECs that migrated on PLL-coated inserts also had similar morphologies and membrane protrusions (data not shown).

To determine if the levels of α7 integrin expression on OECs altered their migratory behaviour on laminin, we counted all the nuclei remaining on the underside of the transwell inserts treated with laminin or PLL. Because some baseline migration of OECs was observed on the neutral PLL substrate, we normalized the results of OEC migration on laminin to the baseline migration levels measured on PLL. Thus, the number of nuclei present on laminin inserts from each α7 integrin genotype was divided by the number of nuclei on the matched PLL inserts and expressed as the “migratory-potential ratio” within each genotype (See Methods). On average, twice as many *α7*^*+/+*^ OECs migrated on laminin compared with those on the PLL substrate (mean migratory potential = 2.14 ± 0.76, mean ± SEM, n = 4). The *α7*^*lacZ/+*^ OECs showed some preference for laminin (migratory potential = 1.45 ± 0.23, n = 4), while *α7*^*lacZ/lacZ*^ OECs had no preference (0.92 ± 0.19, n = 4). Thus significantly more *α7*^*+/+*^ than *α7*^*lacZ/lacZ*^ OECs migrated on laminin (*p*<0.01, Fig 7G); this is consistent with the view that α7 integrin mediates adult OEC migration on laminin.

## Discussion

Using mice that express β-gal in lieu of α7 integrin, we show that α7/β-gal colocalizes with established OEC markers (SOX10, S100β, and AQP1) but not with OMP, a marker for mature olfactory receptor neurons. These results demonstrate that OECs express α7 integrin, the laminin receptor. While the loss of α7 integrin did not change OEC-induced neurite outgrowth in our neuron-OEC co-cultures, OEC motility on laminin-coated transwells was decreased. The migratory potential of wildtype OECs in our motility assay was twice that of *α7*^*lacZ/lacZ*^ OECs. From these findings we conclude that α7 integrin has an important role in laminin-induced migration of adult OECs, but does not directly modulate neurite regeneration *in vitro* after the neurons are dissociated.

### Compensatory integrin binding

Both Flintoff-Dye et al. [27] and Velling et al. [21] report the presence of α7 integrin expression in olfactory bulbs during embryonic development, and we have now identified α7 integrin in adult OECs. Because there were no obvious defects in adult *α7*^*lacZ/lacZ*^ lamina propria or ONL, the initial OEC migration from the olfactory placode into the olfactory bulb can occur in the absence of α7β1 integrin, perhaps because other integrins compensate when the α7 subtype is lost. Specifically, α6 integrin reportedly plays a large role in the developmental organization of the olfactory bulb [45], and Flintoff-Dye et al. [27] found that α6 integrin was up-regulated in various tissues of *α7*^*lacZ/lacZ*^ mice. It is therefore likely that the overall morphological development of *α7*^*lacZ/lacZ*^ lamina propria and ONL is preserved through compensatory mechanisms of integrin binding.

### α7 integrin in neuronal regeneration

Mammalian CNS neurons have a lower intrinsic ability to regenerate following injury than peripheral neurons, and the properties of the glia that surround injured neurons can contribute to their regenerative capacity. In the peripheral nervous system, Schwann cells express α7 integrin, and after trauma both α7 integrin and laminin expression levels increase at the injury site [23, 28]. In contrast, CNS injuries do not increase levels of α7 integrin or its ligand, and regeneration is limited in these areas [24, 46]. One promising CNS therapy for spinal cord injury involves the transplantation of OECs around the injury site, as OECs improve functional recovery following a complete spinal cord transection or a naturally occurring spinal cord injury [5–9]. The mechanisms by which OECs induce functional regeneration are not yet understood. Our results suggest that α7β1 integrin expression on adult OECs may facilitate their extensive migratory ability (Fig 7) but seems not to affect neurite outgrowth directly (Fig 6), since no differences in outgrowth were detected for mouse cortical neurons with and without α7 integrin expression.

### Integrins and glial migration

In our migration assay a baseline level of migration was detected for all genotypes tested on PLL alone, and this finding implies that migratory mechanisms are present that do not involve laminin. Multiple migratory mechanisms are consistent with reports in which neurotrophic factors such as glial cell line-derived neurotrophic factor (GDNF) are reported to stimulate OEC migration [47, 48]. OEC motility is characterized by “lamellipodial waves” representing protrusions of OEC plasma membrane that seem to direct cell-to-cell interactions and migration [48]. Windus et al. [48] reported that GDNF greatly enhances the activity of the OEC waves, while selective inhibitors of JNK and SRC kinases decrease wave formation.

Moreover, Windus et al. [49] reported functionally heterogeneous subtypes of OECs, with differences in their lamellipodial waves and subsequent cell-cell interactions. Specifically, the peripheral OECs from the lamina propria use their lamellipodia to adhere to each other and form strong cell-cell contacts, while central OECs do not adhere until they mature and never form contacts to the extent seen with peripheral OECs. Differential expression of β-DG in the olfactory system, with more extensive reactivity associated with peripheral OECs than on central OECs, may reflect these distinct OEC functions and can be included in the growing list of antigenic markers that define OEC subtypes [1, 4, 50]. Interestingly, OEC expression of β-DG and α7 integrin may parallel differences seen in Schwann cell subtypes; Gardiner et al. [28] reported that β-DG expression was limited to myelinating Schwann cells, while α7 integrin localized to both myelinating and nonmyelinating Schwann cells.

OECs and their close Schwann cell relatives are both derived from the neural crest [31, 51], share numerous surface and intracellular markers, and are implicated in nerve regeneration [24]. The presence of α7 integrin on Schwann cells is well established [23, 28], but our experiments are the first to confirm α7 integrin expression on adult OECs. While expression of α7β1 integrin in adult OECs appears not to facilitate cortical neurite outgrowth, it is likely to participate in the migration of adult OECs *in vivo*.

## Supporting Information

S1 Fig β-dystroglycan expression in the olfactory nerve and olfactory nerve layer of the olfactory bulb. A-C: Antibodies against β-dystroglycan (A, B, green) show immunoreactivity primarily in the olfactory nerve (ON) and olfactory nerve layer (ONL), areas that contain many OECs. Anti-brain lipid-binding protein (BLBP, B, C, red) marks areas that contain large numbers of OECs (arrowheads). OEC expression of β-DG is strongest in the olfactory nerve and where the nerve enters into the ONL. Scale A-C: 50 μm.
(TIF)

S1 Table Mean Total Neurite Length without Normalization. Ten random fields of neurons were photographed from each well, and their neurites were traced with Neurolucida software. The total length of outgrowth per field was summed, and this value was divided by the number of neurons bearing processes to yield the average total neurite outgrowth per neuron. This was repeated for each genotype, two wells per genotype for each of the 3 culture dates. Values are means ± standard error before these results were normalized and plotted in Fig 6I. A total of 6042 neurons were traced with a mean of 755 neurons in the eight experimental groups.
(DOC)

S2 Table Mean Length of Individual Neurites sorted by OEC interaction. Each neurite was scored depending on the type of interaction it made with an OEC: 1) aligned with OECs, 2) crossed an OEC process, or 3) no contact. After sorting, individual processes were averaged and then compared to those grown on laminin, PLL, and PLL with OECs for each interaction type in Fig 6J. No differences were detected between neurites that aligned with *α7*^*+/+*^ or *α7*^*lacZ/lacZ*^ OECs. Neurites that aligned with OECs grew to similar lengths as those that extended on laminin and were significantly longer than processes that crossed or did not contact OECs. Neurites that did not make contact with OECs extended to lengths similar to those grown on PLL alone.
(DOC)
