# Supplementary figures and images for: Olfactory Ensheathing Cells Express α7 Integrin to Mediate Their Migration on Laminin
Source: PLoS One. 2016 Apr 14;11(4):e0153394. doi: 10.1371/journal.pone.0153394 (PMC4831794; doi:10.1371/journal.pone.0153394)

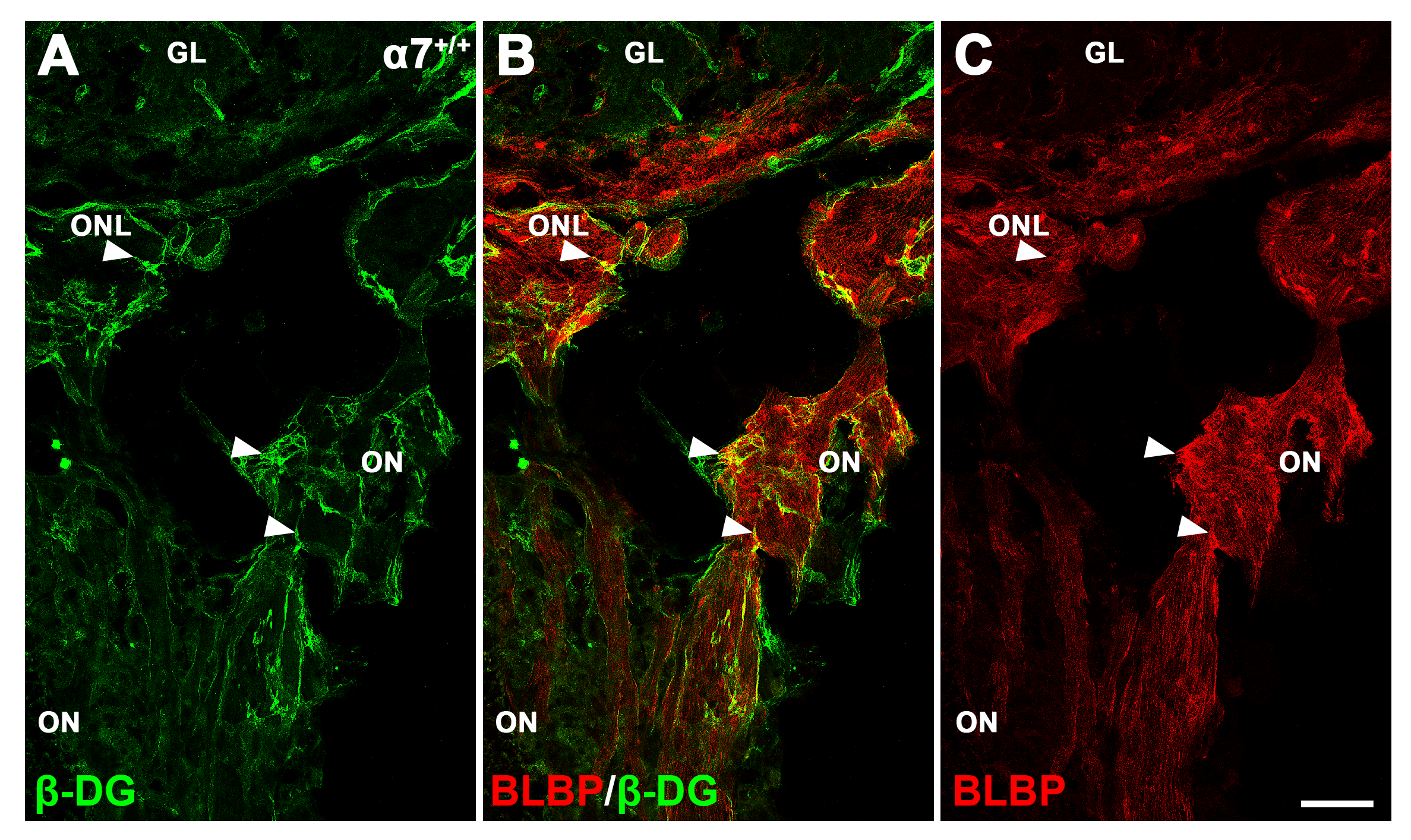

Supplement: S1 Fig — A-C: Antibodies against β-dystroglycan (A, B, green) show immunoreactivity primarily in the olfactory nerve (ON) and olfactory nerve layer (ONL), areas that contain many OECs. Anti-brain lipid-binding protein (BLBP, B, C, red) marks areas that contain large numbers of OECs (arrowheads). OEC expression of β-DG is strongest in the olfactory nerve and where the nerve enters into the ONL. Scale A-C: 50 μm. (TIF) [file pone.0153394.s001.tif]
