# Supplementary material for: Olfactory Ensheathing Cells Express α7 Integrin to Mediate Their Migration on Laminin
Source: PLoS One. 2016 Apr 14;11(4):e0153394. doi: 10.1371/journal.pone.0153394 (PMC4831794; doi:10.1371/journal.pone.0153394)
Supplement: S1 Table — Ten random fields of neurons were photographed from each well, and their neurites were traced with Neurolucida software. The total length of outgrowth per field was summed, and this value was divided by the number of neurons bearing processes to yield the average total neurite outgrowth per neuron. This was repeated for each genotype, two wells per genotype for each of the 3 culture dates. Values are means ± standard error before these results were normalized and plotted in Fig 6I. A total of 6042 neurons were traced with a mean of 755 neurons in the eight experimental groups. (DOC) [file pone.0153394.s002.doc]

|  | **Laminin** | **PLL** | **PLL + *α7^+/+^* OECs** | **PLL + *α7^lacZ/lacZ^* OECs** |
| --- | --- | --- | --- | --- |
| ***α7^+/+^* Neurons** | 264 ± 55 | 130 ± 27 | 198 ± 39 | 224 ± 43 |
| ***α7^lacZ/lacZ^* Neurons** | 224 ± 39 | 113 ± 16 | 195 ± 37 | 201 ± 43 |

**S1 Table: Mean Total Neurite Length without Normalization (μm ± SEM)**
