# Supplementary material for: Olfactory Ensheathing Cells Express α7 Integrin to Mediate Their Migration on Laminin
Source: PLoS One. 2016 Apr 14;11(4):e0153394. doi: 10.1371/journal.pone.0153394 (PMC4831794; doi:10.1371/journal.pone.0153394)
Supplement: S2 Table — Each neurite was scored depending on the type of interaction it made with an OEC: 1) aligned with OECs, 2) crossed an OEC process, or 3) no contact. After sorting, individual processes were averaged and then compared to those grown on laminin, PLL, and PLL with OECs for each interaction type in Fig 6J. No differences were detected between neurites that aligned with α7+/+ or α7lacZ/lacZ OECs. Neurites that aligned with OECs grew to similar lengths as those that extended on laminin and were significantly longer than processes that crossed or did not contact OECs. Neurites that did not make contact with OECs extended to lengths similar to those grown on PLL alone. (DOC) [file pone.0153394.s003.doc]

**S2 Table:**

**Mean Length of Individual Neurites sorted by OEC interaction (µm ± SEM)**

| **Laminin** | 91 ± 13 | **PLL** | 49 ± 7 | **Laminin vs PLL** | *p*= 0.0066 |
| --- | --- | --- | --- | --- | --- |
| ***α7*^+/+^ OECs:**  Aligned | 92 ± 17 | ***α7*^+/+^ OECs:**  Crossed  No contact | 60 ± 11  49 ± 8 | ***α7*^+/+^ OECs:**  Aligned vs PLL  Aligned vs Cross  Aligned vs No contact | *p*= 0.0294  *p*= 0.1038  *p*= 0.0285 |
| ***α7*^lacZ/lacZ^ OECs:**  Aligned | 97 ± 17 | ***α7*^lacZ/lacZ^ OECs:**  Crossed  No contact | 60 ± 14  38 ± 6 | ***α7*^lacZ/lacZ^ OECs:**  Aligned vs PLL  Aligned vs Cross  Aligned vs No contact | *p*= 0.0118  *p*= 0.0477  *p*= 0.0021 |
